# Patient-reported outcomes are superior in patients with Type 2 diabetes treated with liraglutide as compared with exenatide, when added to metformin, sulphonylurea or both: results from a randomized, open-label study

**DOI:** 10.1111/j.1464-5491.2011.03276.x

**Published:** 2011-06

**Authors:** W E Schmidt, J S Christiansen, M Hammer, M J Zychma, J B Buse

**Affiliations:** Department of Medicine I, St Josef Hospital, Ruhr University of Bochum Medical SchoolBochum, Germany; * Department of Endocrinology and Diabetes, Aarhus University HospitalAarhus; † Novo Nordisk A/SBagsvaerd, Denmark; ‡ Department of Medicine, University of North Carolina School of MedicineChapel Hill, NC, USA

**Keywords:** exenatide, liraglutide, patient-reported outcomes, Type 2 diabetes

## Abstract

**Aims:**

The Liraglutide Effect and Action in Diabetes 6 trial was an open-label trial comparing liraglutide with exenatide as an ‘add-on’ to metformin and/or sulphonylurea.

**Methods:**

Patients with Type 2 diabetes were randomized to liraglutide 1.8 mg once daily or exenatide 10 μg twice daily for 26 weeks. This was followed by a 14-week extension phase, in which all patients received liraglutide 1.8 mg once daily.

**Results:**

Patient-reported outcomes were measured in 379 patients using Diabetes Treatment Satisfaction Questionnaire status (DTSQs) and DTSQ change (DTSQc). The change in overall treatment satisfaction (DTSQs score) from baseline at week 26 with liraglutide was 4.71 and with exentaide was 1.66 [difference between groups 3.04 (95% CI 1.73–4.35), *P* < 0.0001]. Five of the six items on the DTSQs improved significantly more with liraglutide than with exenatide (differences: current treatment 0.37, *P* = 0.0093; convenience 0.68, *P* < 0.0001; flexibility 0.57, *P* = 0.0002; recommend 0.49, *P* = 0.0003; continue 0.66, *P* = 0.0001). Patients perceived a greater reduction in hypoglycaemia at week 26 with liraglutide than with exenatide [difference in DTSQc score 0.48 (0.08–0.89), *P* = 0.0193] and a greater reduction in perceived hyperglycaemia [difference 0.74 (0.31–1.17), *P* = 0.0007]. During the extension phase, when all patients received liraglutide, DTSQs scores remained stable in patients who continued on liraglutide and increased significantly (*P* = 0.0026) in those switching from exenatide.

**Conclusions:**

These results demonstrate significant improvements in patients’ treatment satisfaction with liraglutide compared with exenatide.

## Introduction

The negative impact of Type 2 diabetes on health status is well established [1]. A number of factors contribute towards changes in patient-reported outcome measures for people with Type 2 diabetes.These include:

- poor glycaemic control, associated with worsening of symptom distress, general perceived health and cognitive functioning [2];
- overweight or obesity, which has an adverse effect on mobility, self-care and usual activities and causes pain/discomfort [3,4];
- treatment side effects such as hypoglycaemia, which can affect all domains/sections of the measure of functional health and well-being (Short Form 36) [5];
- the presence of diabetes complications, which is associated with depression, fatigue, less vigour, and reduced mobility, self-care and usual activities [6].

Improvements in these factors will generally translate into improved health status [7]. In addition to measuring the clinical efficacy and safety of any novel anti-diabetes drug, patient-reported outcome measures are important to assess the patients’ perceptions of their condition and the benefits of treatment. Thus, patient-reported outcome measures may be important in complementing clinical measures of treatment efficacy and safety.

Glucagon-like peptide 1 (GLP-1) analogues represent a new class of anti-diabetes medications that, through their glucose-dependent mechanism of action, may avoid some of the limitations of earlier-generation agents. They are not associated with oedema and have a low incidence of hypoglycaemia. In addition to providing glucoregulatory effects, weight loss is common among patients receiving GLP-1 analogues [8]. In a recent consensus statement by the American Diabetes Association/European Association for the Study of Diabetes, agonists of the GLP-1 receptor are listed among the agents that can be added to metformin therapy when weight loss is important [9].

Two agonists of the GLP-1 receptor have been approved for treating Type 2 diabetes. Exenatide (Eli Lilly and Company, Indianapolis, IN, USA) requires twice-daily subcutaneous administration and has demonstrated efficacy with respect to glycaemic control and weight loss [9,10]. Liraglutide (Novo Nordisk, Bagsværd, Denmark) is a human GLP-1 agonist, which is effective with once-daily administration, can be injected without regard to timing of meals, and is associated with weight loss and a low risk of hypoglycaemia [8,10–13]. The risk of hypoglycaemia and weight gain have been recognized as important factors in making treatment decisions [14] and reductions may improve health status. Data from a trial comparing liraglutide with glimepiride suggest that treatment with liraglutide improves patient-reported outcomes and weight assessments compared with glimepiride [15].

The Liraglutide Effect and Action in Diabetes 6 (LEAD 6) trial was a large, phase-IIIb clinical trial comparing the efficacy and safety of either liraglutide or exenatide in combination with metformin and/or sulphonylurea in Type 2 diabetes. Greater reductions in HbA_1c_ (*P* < 0.0001), a lower incidence of hypoglycaemia and similar reductions in weight were observed with liraglutide 1.8 mg once daily compared with exenatide 10 μg twice daily [16]. Patient-reported outcome measures were assessed in LEAD 6 by the Diabetes Treatment Satisfaction Questionnaire (DTSQ) [17] to obtain information on treatment satisfaction with liraglutide and exenatide. The results from these patient-reported outcome measure analyses are reported here.

## Methods

The methodology of LEAD 6 is summarized here and described fully elsewhere [16]. LEAD 6 was conducted at 132 sites in 15 countries, in accordance with the Declaration of Helsinki and its amendments, and was approved by all relevant health authorities. All participants in the trial gave written informed consent before taking part.

### Patients

LEAD 6 included patients aged 18–80 years with Type 2 diabetes who were receiving a stable regimen of metformin, sulphonylurea or both for at least 3 months, and had HbA_1c_ values of 7.0–11.0% (53–97 mmol/mol) and a BMI ≤ 45 kg/m^2^. Patients had no previous treatment with insulin, exenatide or liraglutide.

### Study design

**FIGURE 1:**
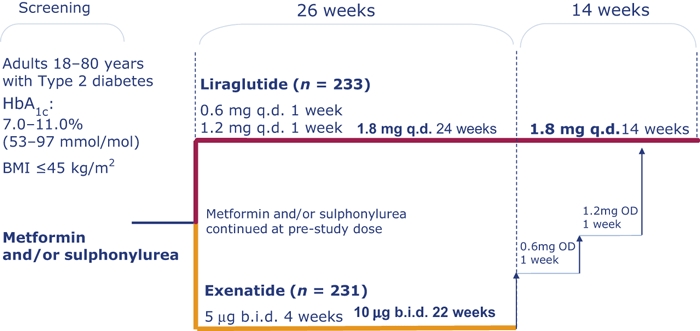
Study design of Liraglutide Effect and Action in Diabetes 6 (LEAD 6).

The trial was a randomized, open-label, parallel-group comparison of liraglutide 1.8 mg once daily with exenatide 10 μg twice daily for 26 weeks. Both treatments were added to an existing treatment regimen that included metformin, sulphonylurea or both (Fig. 1). After randomization, liraglutide was escalated to the 1.8-mg dose in weekly 0.6-mg increments (i.e. the starting dose of 0.6 mg was increased after 1 week to 1.2 mg and after 2 weeks to 1.8 mg) and exenatide was escalated up to the 10-μg dose after 4 weeks of 5 μg twice daily. Patients then remained on these maximum recommended doses for 22–24 weeks. This was followed by an ongoing non-randomized extension phase with endpoint assessment at 14 weeks, during which all patients received liraglutide 1.8 mg once daily; so patients on exenatide 10 μg twice daily were switched to liraglutide 1.8 mg once daily (Fig. 1). The study, which began on 24 August 2007, was completed on 9 April 2008.

### Assessments

During the trial, patients were assessed for efficacy and safety variables. Efficacy variables included HbA_1c_, weight, waist and hip circumference, fasting plasma glucose, self-measured fasting plasma glucose and 7-point glucose profile, B-cell function and glucagon, fasting lipid profile, blood pressure, cardiovascular biomarkers and patient-reported outcome measures. Safety variables included physical examination, pulse rate and hypoglycaemic episodes; laboratory tests included haematological and biochemical measures, and measurement of antibodies to liraglutide and exenatide.

Patient-reported outcomes were assessed using a validated self-administered questionnaire, DTSQ [17], in eight of the participating countries, which included Austria, Denmark, Finland, Germany, Ireland, Poland, Romania and the USA. The remaining countries were not included in the patient-reported outcome sub-study because of lack of available linguistic validated patient-reported outcome measures. The DTSQ consists of two versions, both assessing treatment satisfaction among patients, as well as the perceived frequency of hyperglycaemia and hypoglycaemia. The DTSQs (status version) measures status at a given time point and the DTSQc (change version) measures changes over a period of time (i.e. from baseline). Both of these versions of the DTSQ assess treatment satisfaction by six items on ‘current treatment’, ‘convenience’, ‘flexibility’, ‘understanding’, ‘recommend’ and ‘continue’. Perceived frequency of hyperglycaemia and hypoglycaemia were measured by one item each. The individual questions on the DTSQ were scored on a 7-point Likert scale, from 0 to 6 in the DTSQs and from –3 to +3 in the DTSQc. Scores for the six treatment satisfaction items were summed to give a total score of 0 to 36 for the DTSQs and –18 to +18 for the DTSQc. A higher score denotes greater patient satisfaction. The perceived frequency of hyperglycaemia and hypoglycaemia items scores range from 0 (‘none of the time’) to 6 (‘most of the time’) in the DTSQs and from –3 (‘much less of the time now’) to +3 (‘much more of the time now’) in the DTSQc.

The status version (DTSQs) was completed by patients at baseline (randomization), week 26, week 34 and week 40 and at early withdrawal from the study; the change version (DTSQc) was completed by patients at week 26 and at week 34 (for perception of change from baseline until week 26 and from week 26 to week 34) [17,18].

### Statistical analyses

The detailed statistical analyses of efficacy endpoints of LEAD 6 are described elsewhere [16]. The primary endpoint was analysed using both the intention-to-treat and the per-protocol populations, but patient-reported outcome analyses were restricted to the intention-to-treat population (all randomized participants who received at least one dose of the study drugs). Missing data were replaced using the last-observation-carried-forward approach, but for DTSQc data (which were collected at week 26 only) observed cases only were included. For the patient-reported outcome analyses, DTSQs endpoints were analysed at week 26 using the standard ANCOVA model with treatment, country and previous treatment as fixed effects, and DTSQc endpoints were analysed using an ANCOVA model with treatment, country and previous treatment as fixed effects and baseline DTSQs as covariate.

## Results

**FIGURE 2:**
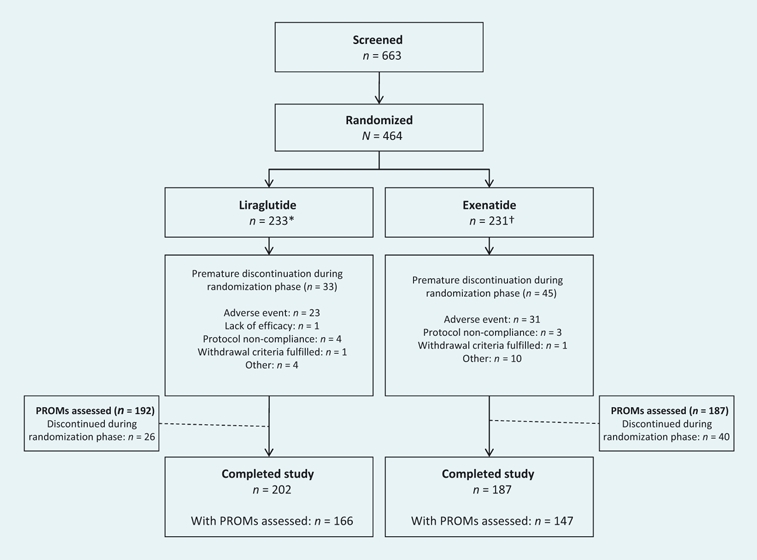
Patient disposition from screening until the end of the randomization phase (26 weeks) in Liraglutide Effect and Action in Diabetes 6 (LEAD 6). *Intention-to-treat (ITT), per-protocol (PP) and safety populations in the liraglutide treatment group were 233, 193 and 235, respectively. †ITT, PP and safety populations in the exenatide treatment group were 231, 172 and 232, respectively. PROMs, patient-reported outcome measures.

In LEAD 6, 464 patients were randomized and exposed to treatment (intention-to-treat population); 233 received liraglutide and 231 received exenatide in addition to their existing treatment regimen [16] (Fig. 2). Patient-reported outcome measures were assessed in 379 patients (82%). In the full study population, 78 patients (33 in the liraglutide group and 45 in the exenatide group) failed to complete the randomization portion of the study and were withdrawn (before entering the extension phase), primarily because of adverse events (Fig. 2). Of these withdrawals, 26 patients in the liraglutide group and 40 patients in the exenatide group were in the subset of patients assessed for patient-reported outcome measures and completed a follow-up DTSQ at the time of withdrawal, but not at week 26 or subsequent visits.

The two treatment groups, the population in the main study and the patient-reported outcome-assessed subgroup population, were well matched in their baseline characteristics (Table 1). Most patients were receiving a metformin/sulphonylurea combination at enrolment, and these patients had a longer mean duration of diabetes (9.5 years) than patients receiving monotherapy with either drug (5.3 and 7.5 years for those receiving metformin and sulphonylurea monotherapy, respectively). The DTSQs scores indicated very similar baseline levels of treatment satisfaction in the two randomized treatment groups.

**Table 1:** Baseline patient and disease characteristics of the LEAD 6 ITT population and the subgroup of patients that completed the DTSQ

	ITT population		PRO-assessed population	
		
Variable	Liraglutide group	Exenatide group	Liraglutide group	Exenatide group
Men/women, n	114/119	127/104	94/98	99/88
Mean (sd) age, years	56.3 (9.8)	57.1 (10.8)	56.8 (10.0)	56.6 (11.1)
Mean (sd) weight, kg	93.1 (20.1)	93.0 (19.5)	93.8 (20.6)	95.0 (20.4)
Mean (sd) BMI, kg/m^2^	32.9 (5.5)	32.9 (5.7)	33.1 (5.7)	33.5 (5.7)
Mean (sd) duration of diabetes, years	8.5 (6.2)	7.9 (5.9)	8.6 (6.3)	7.6 (5.9)
Mean (sd) HbA_1c_, %	8.2 (1.0)	8.1 (1.0)	8.2 (1.0)	8.1 (1.0)
Previous anti-diabetic treatment, *n*				
Metformin	64	63	52	57
Sulphonylurea	24	21	17	14
Metformin + sulphonylurea	145	147	123	116
Mean (sd) fasting blood glucose, mmol/l	9.8 (2.5)	9.5 (2.4)	9.8 (2.6)	9.6 (2.5)
DTSQs score	NA	NA	27.4	27.6

BMI, body mass index; DTSQ, Diabetes Treatment Satisfaction Questionnaire; ITT, intention-to-treat; LEAD 6, Liraglutide Effect and Action in Diabetes 6; NA, not applicable; PRO, patient-reported outcome.

### Patient-reported outcome measures

**FIGURE 3:**
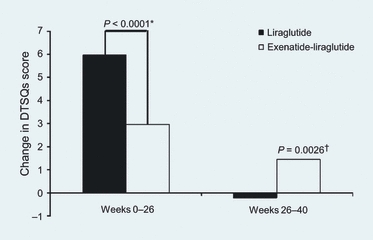
Overall treatment satisfaction, as measured by the Diabetes Treatment Satisfaction Questionnaire status version (DTSQs), in patients initially receiving liraglutide 1.8 mg once daily or exenatide 10 μg twice daily for 26 weeks, and then receiving liraglutide 1.8 mg once daily for 14 weeks (patient-reported outcome analysis population with data in extension phase of trial). *Comparison of liraglutide vs. exenatide change from baseline. †Comparison of week 40 vs. week 26 in the exenatide to liraglutide group.

The results of LEAD 6 are presented in more detail elsewhere [16]. The overall treatment satisfaction, as measured by the DTSQs, was similar in the two treatment groups at baseline, but, after 26 weeks of treatment, the DTSQs score increased more in the group receiving liraglutide than the group receiving exenatide [from 27.4 to 32.1 in the liraglutide group, absolute change 4.71; and from 27.6 to 29.3 in the exenatide group, absolute change 1.66; intention-to-treat–last observation-carried-forward population; difference between groups 3.04 (95% CI 1.73–4.35), *P* < 0.0001]. The proportion of patients that were satisfied overall with their treatment (predefined as an overall score on the DTSQs of > 24) was 91% in the liraglutide group and 82% in the exenatide group [difference between groups 9.0% (95% CI 1.2–16.7%), *P* = 0.0192]. The DTSQc score, which measured the change from baseline in a single score, confirmed that levels of treatment satisfaction were higher in the liraglutide group (mean score 15.18) than in the exenatide group (mean score 13.30; *P* = 0.0004 observed cases), and the proportion of ‘satisfied’ patients (defined as an overall score on the DTSQc of > 6) was 94% in the liraglutide group and 86% in the exenatide group [difference between groups 8.5% (95% CI 1.1–15.9%), *P* = 0.0176]. During the extension phase of the trial, when all patients switched to liraglutide 1.8 mg therapy, DTSQs scores remained stable in patients who had received liraglutide in the first 26 weeks of the trial and increased significantly (*P* = 0.0026) at week 40 in those who switched from exenatide to liraglutide at week 26 (Fig. 3 and Table 3). The DTSQc scores also showed improvements from week 26 to week 34 in patients switched from exenatide treatment to liraglutide treatment.

**Table 3:** Mean (sd) change from week 26 in DTSQ scores (patient-reported outcome analysis population with data in extension phase of trial) to end of week 40 (DTSQs) or end of week 34 (all patients received liraglutide)

	DTSQs			DTSQc		
		
Item	Liraglutide†	Exenatide†	Relative difference between treatments (95% CI; *P*-value*)	Liraglutide†	Exenatide†	Relative difference between treatments (95% CI; *P*-value*)
Current treatment						
Change from week 26	−0.10 (0.91) (*n* = 160)	0.10 (0.95) (*n* = 134)	−0.20 (−0.42 to 0.02; 0.070)	−0.08 (0.78) (*n* = 155)	−0.02 (1.14) (*n* = 131)	−0.06 (−0.29 to 0.17; 0.582)
Convenience						
Change from week 26	−0.06 (1.04) (*n* = 161)	0.38 (1.39) (*n* = 136)	−0.47 (−0.75 to −0.19; 0.001)	0.05 (1.03) (*n* = 153)	0.11 (1.55) (*n* = 131)	−0.08 (−0.40 to 0.23; 0.592)
Flexibility						
Change from week 26	−0.07 (1.38) (*n* = 161)	0.29 (1.44) (*n* = 137)	−0.31 (−0.63 to 0.02; 0.064)	−0.02 (1.18) (*n* = 153)	0.24 (1.63) (*n* = 131)	−0.23 (−0.56 to 0.10; 0.173)
Understanding						
Change from week 26	0.09 (0.86) (*n* = 160)	0.15 (0.92) (*n* = 136)	−0.05 (−0.25 to 0.16; 0.660)	−0.04 (1.03) (*n* = 154)	−0.03 (0.94) (*n* = 131)	−0.01 (−0.24 to 0.22; 0.921)
Recommend						
Change from week 26	0.01 (0.81) (*n* = 160)	0.16 (1.18) (*n* = 134)	−0.17 (−0.39 to 0.05; 0.138)	−0.04 (1.04) (*n* = 153)	0.14 (1.07) (*n* = 129)	−0.18 (−0.44 to 0.07; 0.160)
Continue						
Change from week 26	−0.01 (0.69) (*n* = 161)	0.26 (1.22) (*n* = 135)	−0.29 (−0.51 to −0.06; 0.013)	−0.01 (0.72) (*n* = 152)	0.08 (1.36) (*n* = 129)	−0.09 (−0.35 to 0.17; 0.499)
Perceived hyperglycaemia						
Change from week 26	−0.31 (1.60) (*n* = 160)	−0.50 (1.92) (*n* = 137)	0.23 (−0.18 to 0.64; 0.264)	−0.01 (2.14) (*n* = 154)	−0.44 (1.97) (*n* = 131)	0.48 (−0.01 to 0.98; 0.056)
Perceived hypoglycaemia						
Change from week 26	−0.21 (1.74) (*n* = 161)	−0.07 (1.52) (*n* = 136)	−0.11 (−0.49 to 0.27; 0.582)	−0.07 (2.29) (*n* = 155)	−0.34 (2.04) (*n* = 131)	0.34 (−0.17 to 0.86; 0.191)

* Versus week 26 data.

† Treatment received during the first 26 weeks of the trial.

DTSQc, Diabetes Treatment Satisfaction Questionnaire change version; DTSQs, Diabetes Treatment Satisfaction Questionnaire status version.

The six items of the DTSQ were analysed, and the change in DTSQs score from baseline to week 26 was greater in all aspects of treatment satisfaction (‘satisfaction with treatment’, ‘convenience’, ‘flexibility’, ‘recommend’ and ‘continue’) with liraglutide than with exenatide, and all differences were statistically significant, except for the item measuring ‘satisfied with diabetes understanding’ (Table 2). Results from the DTSQc (observed cases) also showed greater improvements in all items with liraglutide than with exenatide, except the item measuring ‘diabetes understanding’, which was similar in the two treatment groups (Table 2).

**Table 2:** Mean (sd) change from baseline in DTSQ scores (ITT)† to end of week 26

	DTSQs			DTSQc		
		
Item	Liraglutide	Exenatide	Relative difference between treatments (95% CI; *P*-value*)	Liraglutide	Exenatide	Relative difference between treatments (95% CI; *P*-value*)
Current treatment						
Baseline	4.4 (1.7) (*n* = 188)	4.6 (1.4) (*n* = 187)				
Change from baseline	1.01 (1.99) (*n* = 179)	0.49 (1.82) (*n* = 172)	0.37 (0.09–0.65; 0.009)	2.72 (0.69) (*n* = 164)	2.39 (0.93) (*n* = 144)	0.35 (0.17–0.53; 0.0002)
Convenience						
Baseline	4.8 (1.3) (*n* = 187)	4.7 (1.2) (*n* = 187)				
Change from baseline	0.59 (1.51) (*n* = 179)	0.02 (1.75) (*n* = 174)	0.68 (0.41–0.92; < 0.0001)	2.47 (0.98) (*n* = 164)	2.10 (1.28) (*n* = 144)	0.40 (0.15–0.66; 0.002)
Flexibility						
Baseline	4.5 (1.6) (*n* = 187)	4.4 (1.5) (*n* = 187)				
Change from baseline	0.70 (1.80) (*n* = 179)	0.19 (1.85) (*n* = 175)	0.57 (0.27–0.86; 0.0002)	2.30 (1.05) (*n* = 163)	1.93 (1.34) (*n* = 144)	0.38 (0.12–0.64; 0.005)
Understanding						
Baseline	4.6 (1.3) (*n* = 188)	4.6 (1.3) (*n* = 187)				
Change from baseline	0.49 (1.19) (*n* = 179)	0.38 (1.52) (*n* = 174)	0.14 (−0.09 to 0.36; 0.236)	2.36 (0.85) (*n* = 163)	2.33 (0.91) (*n* = 144)	0.02 (−0.17 to 0.21; 0.828)
Recommend						
Baseline	4.6 (1.6) (*n* = 186)	4.8 (1.4) (*n* = 185)				
Change from baseline	0.96 (1.64) (*n* = 178)	0.35 (1.85) (*n* = 170)	0.49 (0.23–0.75; 0.0003)	2.67 (0.90) (*n* = 163)	2.41 (1.02) (*n* = 143)	0.28 (0.06–0.50; 0.012)
Continue						
Baseline	4.4 (1.8) (*n* = 186)	4.5 (1.7) (*n* = 186)				
Change from baseline	1.03 (2.25) (*n* = 178)	0.25 (2.35) (*n* = 173)	0.66 (0.33–0.99; 0.0001)	2.71 (0.74) (*n* = 163)	2.32 (1.14) (*n* = 143)	0.40 (0.19–0.62; 0.0003)
Perceived hyperglycaemia						
Baseline	3.8 (1.8) (n = 185)	3.8 (1.8) (*n* = 186)				
Change from baseline	−1.82 (2.16) (*n* = 176)	−1.61 (2.29) (*n* = 174)	−0.27 (−0.63 to 0.09; 0.142)	−0.99 (1.86) (*n* = 163)	−0.33 (1.93) (*n* = 145)	−0.74 (−1.17 to −0.31; 0.0007)
Perceived hypoglycaemia						
Baseline	1.0 (1.5) (*n* = 187)	0.9 (1.4) (*n* = 186)				
Change from baseline	0.06 (2.02) (*n* = 179)	0.11 (1.69) (*n* = 173)	−0.02 (−0.32 to 0.29; 0.908)	−0.88 (1.78) (*n* = 164)	−0.44 (1.80) (*n* = 145)	−0.48 (−0.89 to −0.08; 0.019)

* Versus exenatide group.

† DTSQs scores were evaluated by LOCF methodology; DTSQc scores were analysed in the per-protocol population.

DTSQc, Diabetes Treatment Satisfaction Questionnaire change version; DTSQs, Diabetes Treatment Satisfaction Questionnaire status version; ITT, intention to treat; LOCF, last observation carried forward.

Patients receiving liraglutide perceived a greater reduction in hypoglycaemia and hyperglycaemia from baseline to week 26 than patients receiving exenatide, according to the DTSQc but not DTSQs scores (Table 2).

## Discussion

This current analysis of the patient-reported outcome data from LEAD 6 shows that liraglutide is also associated with greater improvements in treatment satisfaction and less perceived hypoglycaemia or hyperglycaemia than exenatide. The extension phase of the trial shows that the level of treatment satisfaction is significantly increased when patients are switched from exenatide treatment to liraglutide treatment.

Glycaemic control in patients receiving oral anti-diabetic drugs for Type 2 diabetes eventually deteriorates and the patients may suffer from frequent hypoglycaemia and weight gain [19]. Treatment with agonists of the GLP-1 receptor reduces HbA_1c_, induces weight loss, has a low risk of hypoglycaemia [9–12] and may therefore provide a useful addition to the available oral ant-diabetic drugs. The patient-reported outcome data demonstrate high rates of patient satisfaction with both exenatide and liraglutide. These data show that patients prefer many aspects of treatment satisfaction (measured by DTSQ), including convenience and flexibility, when receiving liraglutide than when receiving exenatide. A number of factors may contribute towards better patients’ treatment satisfaction with liraglutide: for example, the once-daily dosing may account for improved convenience with liraglutide, as opposed to the twice-daily dosing before meals with exenatide; and improved flexibility with liraglutide may reflect the fact that daily dosing can be administered independently of time of day or meals, as long as it is administered approximately every 24 h. Data derived from a quality of life questionnaire have emphasized the important contribution of dietary freedom to overall quality of life [20]. In addition, treatment satisfaction may be driven by the greater reductions in fasting glucose, HbA_1c_ levels and less frequent hypoglycaemia with liraglutide treatment. Adverse events were reported in 74.9% and 78.9% of patients with liraglutide and exenatide, respectively, in LEAD 6, and nausea was more frequent and more persistent with exenatide than with liraglutide, and this may also influence treatment satisfaction. Along with weight loss, patient satisfaction may improve adherence to treatment and, thus, these patient-reported outcome data may be an important contribution to clinical decisions.

The results from the DTSQc scores for perceived hyperglycaemia and hypoglycaemia may indicate that patients feel more in control of their diabetes (i.e. they have more stable glycaemic levels), supported by the significantly fewer hypoglycaemia events and improved glycaemic control (as measured by HbA_1c_ and fasting plasma glucose) with liraglutide than exenatide.

The comparator drug in this study, exenatide, was previously compared with insulin glargine, where the DTSQ was included to assess the patients’ treatment satisfaction [21]. The results showed that exenatide and insulin glargine both improved treatment satisfaction, but that there was no difference between treatment groups after 26 weeks of treatment [21]. Recently, the long-acting formulation of exenatide given once weekly has been shown to provide better glycaemic control than twice-daily exenatide [22]. In that head-to-head study (a 30-week, open-label trial that included 295 patients with Type 2 diabetes), patients’ treatment satisfaction was also measured by the DTSQ and, although the results showed a trend towards greater treatment satisfaction with exenatide once weekly vs. twice daily, the difference was not statistically significant [7].

While the open-label design of this study limits the full interpretation of the findings, there are many reasons to believe that the results reported here are reliable. LEAD 6 is a large, well-controlled study with a high proportion of patients participating in patient-reported outcome assessments. As patient-reported outcome assessment adds an additional level of complexity to the clinical trial process, the number of patient-reported outcome assessments was limited to baseline (at randomization) and to the end of the trial (week 26), but assessments in the extension phase have also provided useful longer-term data. As treatment satisfaction was the endpoint of the study, blinding was not feasible: different pens are used to administer liraglutide and exenatide and the two drugs had a different dose frequency and time of administration.

Although patient-reported outcome assessments are subjective and should be interpreted accordingly, the DTSQ is a validated measure for assessing treatment satisfaction and perceived hyperglycaemia and hypoglycaemia [17,18]. The clinical significance of the changes measured in DTSQ is worth discussing. DTSQ is a measure of treatment satisfaction that was designed explicitly to measure issues of importance to patients and therefore any statistically significant differences measured by DTSQ will necessarily be an important difference [23]. Treatment satisfaction has been shown to significantly correlate with the duration of diabetes and perceived blood glucose control using the DTSQ [17] and the reliability of DTSQ has been demonstrated [24]. The DTSQ is highly sensitive to major changes in treatment, for example, from tablets to injections [25], or from conventional (more rigid insulin regimen with fixed meal times) to a more flexible insulin dosing (allowing for dietary freedom) [26]. The DTSQs has been used to measure treatment satisfaction during treatment with injectable insulin [27], but the phenomenon of ceiling and floor effects (when data are skewed, with a proportion of respondents having optimal scores at baseline) may lead to underestimation of treatment effects [28]. However, the use of the DTSQc alongside the DTSQs should overcome this [18] and the results with both measures were consistent in our study. The DTSQ also has proven sensitivity when measuring differences between treatment groups [24].

In conclusion, the clinical benefits of once-daily liraglutide over twice-daily exenatide, when used in combination with metformin, sulphonylurea or both in people with Type 2 diabetes, are accompanied by significant improvements in treatment satisfaction, including the important aspects of convenience, flexibility, perceived hypoglycaemia and overall patient satisfaction.

## Abbreviations

DTSQc: Diabetes Treatment Satisfaction Questionnaire change version
DTSQs: Diabetes Treatment Satisfaction Questionnaire status version
GLP-1: glucagon-like peptide 1
LEAD 6: Liraglutide Effect and Action in Diabetes 6
